# Updated Database and Trends of Declared Low- and No-Calorie Sweeteners From Foods and Beverages Marketed in Spain

**DOI:** 10.3389/fnut.2021.670422

**Published:** 2021-07-29

**Authors:** Mª de Lourdes Samaniego-Vaesken, Beatriz González-Fernández, Teresa Partearroyo, Rafael Urrialde, Gregorio Varela-Moreiras

**Affiliations:** ^1^Departamento de Ciencias Farmacéuticas y de la Salud, Facultad de Farmacia, Universidad San Pablo-CEU, CEU Universities, Urbanización Montepríncipe, Madrid, Spain; ^2^Dietista-Nutricionista, Torres y Carrera, Madrid, Spain; ^3^Departamento de Genética, Fisiología y Microbiología, Facultad de Ciencias Biológicas, Universidad Complutense de Madrid, Madrid, Spain; ^4^Fundación Española de la Nutrición, c/General Álvarez de Castro 20, Madrid, Spain

**Keywords:** food composition database, food groups, Spain, reformulation, low- and no-calorie sweeteners

## Abstract

**Background:** The past few years have witnessed an increase in the availability of food products containing one or more low- and no-calorie sweeteners (LNCS) in the Spanish market, mostly due to the new massive reformulation plan. However, these are not included in food composition tables or databases, and, therefore, assessment of their intake among the population is complex. This study aims to update a database including commercialized foods and beverages.

**Method:** A systematic search of ingredients information from the different food and beverage categories was undertaken during 2019 by recording the availability and type of LNCS declared in the information of the product from labels and online shopping platforms of retailers from Spain to update a previous food composition database compiled in 2017.

**Results:** A total of 1,238 products were identified. The major groups were sugar and sweets (24%), non-alcoholic beverages (21%), cereals and grains (19%), and milk and dairy products (14%) accounting for >70% of total products. The mainly declared LNCS were sorbitol (19.5%), sucralose (19.5%), and acesulfame K (19.2%).

**Conclusion:** There is a wide variety of products that include LNCS as a main ingredient with higher availability than when compared with the results of database of 2017, consequently, it might be expected that LNCS are commonly consumed at present in the Spanish diet.

## Background

Low- and no-calorie sweeteners (LNCS), also known as artificial, non-nutritive, or intense sweeteners, comprise a group of food additives that provide high sweetness intensity per gram of food and beverage products (1). They are used in very small quantities and deliver no or fewer calories, replacing added sugars in a variety of food products (2). The use of LNCS has become more common for manufacturers to develop new products and to comply with food and beverage reformulation practices to decrease energy resulting from added sugars. Furthermore, there is general consumer interest in reducing energy intake (TE), and food products containing LNCS have become more popular choice (2, 3). At present, we can find LNCS as ingredients in products labeled as “sugar-free” or “without added sugars” but also in regular products together with low amount of added sugars.

There are currently 19 compounds authorized by the European regulations for use in food products: sorbitol (E-420), mannitol (E-412), acesulfame K (E-950), aspartame (E-951), cyclamate (E-952), isomalt (E-953), saccharine and its sodium, potassium, and calcium salts (E-954), sucralose (E-955), thaumatin (E-957), neohesperidine DC (E-959), steviol glycosides (E-960), neotame (E-961), salt of aspartame-acesulfame (E-962), polyglycitol syrup (E-964), maltitols (E-965), lactitol (E-966), xylitol (E-967), erythritol (E-968), and advantame (E-969) (4). The use of sweeteners in the European Union (EU) is in accordance with Commission Regulations numbers 231/2012 (5) and 1169/2011 (6), which indicate that food labeling must specify the presence of these additives in the list of ingredients as well as next to the name of the product, “with LNCS” (EU). Therefore, the identified sweeteners have only been at the level of declaration of presence, not of quantity present in the food product.

Previous research has identified the most consumed food groups containing LNCS by a representative sample of the Spanish population aged 9–75 years from the ANIBES study (“anthropometric data, macronutrients, and micronutrients intake, as well as the practice of physical activity, socioeconomic data, and lifestyles of the population”) (7) where we described that from 1,164 products analyzed, 10% contained LNCS in their composition and 5.1% declared a combined use of added sugars and LNCS declared on their labels. LNCS were mainly present in diet soft drinks (100%), chewing gum and candies (89%), soya drinks (45%), and yogurt and fermented milk (18%). For this reason, a comprehensive food composition database was developed in 2017 that surveyed all available products from the Spanish market that declared LNCS in their ingredient lists (8).

The main source of information for consumers and researchers about the presence of LNCS in packaged foods are food labels and, specifically, the ingredients list, which allows their identification. However, manufacturers are not mandated to declare added levels of LNCS, which should always remain under the quantities authorized by the European Food Safety Authority (9). At present, the lack of updated and comprehensive data on the type of LNCS in different food products challenges the assessment and monitorization of LNCS consumption, which remains unavailable in most dietary surveys (10, 11).

In 2017, the Spanish Ministry of Health, Social Services and Equality, through the Spanish Agency for Food Safety and Nutrition (AESAN) launched the “Collaboration plan to improve the composition of food and beverages and other measures 2017–2020” within the framework of the “Strategy of Nutrition, Physical Activity and Prevention of Obesity” (NAOS) of AESAN (12). This Plan included reformulation commitments from different sectors of the manufacture and distribution of food products, mainly foods and drinks that are regularly consumed by children, adolescents, and their families, and it was mainly focused on the reduction of added sugar, sodium, and saturated fats. The plan of AESAN was aimed at improving the nutritional quality of the diet, promoting a healthier food intake to prevent or reduce the incidence of overweight and obesity, and related pathologies. The agreed reformulation measures affected food and beverages belonging to 12 groups: soft drinks, pastries and cakes, breakfast cereals, creams, meat products, cookies, ice cream, fruit nectars, specially packaged bread, ready-to-eat-meals, dairy products, and sauces. In any case, it should be highlighted that food and beverage groups included in the proposed plan are responsible for providing 44.5% of the total daily energy from foods with added sugars in Spain (12). Therefore, the overall aim of the plan of AESAN was to reduce from 5 to 10%, depending on the groups, food contents in added sugars, saturated fats, and salt by the end of 2020 (12). In this regard, the use of LNCS as added sugar substitutes is highly relevant but still has not been assessed. It is, therefore, of importance to monitor potential exposures following the requirement to reduce the level of sugar intake, to ensure there is no shift in intakes, particularly for high-risk individuals, such as diabetics and children with specific dietary requirements (13).

For all the aforementioned, in the present study, we aimed to update a previously existing database designed and compiled by the research group in 2017, including all foods and beverages declaring LNCS commercialized in Spain (8).

## Materials and Methods

### Study Design and Data Collection

This cross-sectional study comprised of a systematic search and screening of label ingredient information of food and beverage product to identify one or more of the EU authorized LNCS, available through three major online retailer shopping platforms at the national level, accounting for ≥85% of the market (14, 15). The presence and type of LNCS declared, which were included in the ingredient lists of available foods and beverages, was identified and recorded, all of these products were processed foods as they contain several ingredients and additives (with the E-number identification). Data was collected throughout 2019, and all the products which declared an LCNS within their ingredient information were included. The acquired information was compiled in an in-house database comprising Microsoft^TM^ Excel 2013 spreadsheets, where each product was coded and classified according to label denomination into nine groups and 28 subgroups. Data including product name, denomination, brand, ingredient list, nutritional information were recorded.

### Identification of LCNS in Packaged Foods, Database Compilation, and Data Management

Low- and no calorie sweeteners were reviewed in the online platform information in accordance with the list of 19 LNCS types authorized by the EU food labeling regulations (EU). Product names, categories, ingredients, and food additives found on the description of each food and beverage were compiled using a Microsoft^TM^ Excel 2013 spreadsheet for analysis. Quality assurance control was performed by two researchers, who checked the data independently. The presence and frequency of the different LCNS were analyzed globally and within each food and beverage subgroup. In addition, the individual or combined use of each LCNS in each food group was also evaluated.

## Results

**Figure 1:**
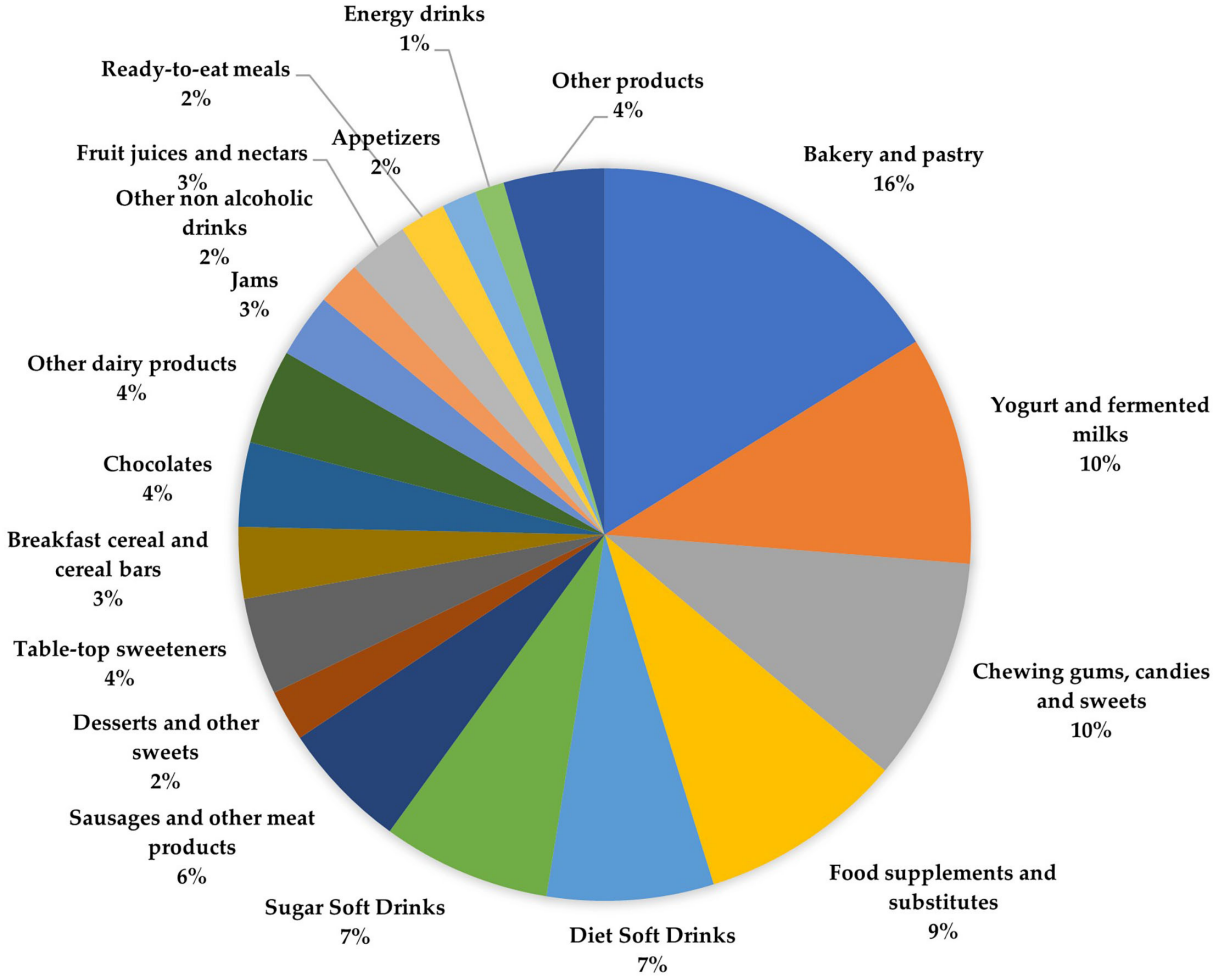
Distribution of food and beverage subgroups (%) available in the Spanish market containing one or more low and no-calorie sweeteners (LCNS) as declared in their ingredient information.

The database was updated and included a total of 1,238 products declaring LNCS in their ingredient lists, which were identified from the survey performed among a representative sample of online food and drink retailers. The major groups identified included the following (Figure 1): sugar and sweets (24%), non-alcoholic beverages (21%), cereals and grains (19%), and milk and dairy products (14%), all of which accounted for more than 70% of total available products in the compiled database.

The different food and beverage groups and subgroups distribution, together with the number of added LNCS is shown in Table 1.

**Table 1:** Distribution of food and beverage groups and subgroups available in the Spanish market (*n*, %), according to the number of declared low and no-calorie sweeteners (LCNS).

**Food groups and subgroups**	**Number of declared LNCS**							
	**1**	**2**	**3**	**4**	**5**	**6**	**7**	**8**
Sugar and sweets (*n* = 299; 24.1%)	97 (32.4%)	88 (29.4%)	54 (18%)	23 (7.7%)	12 (4%)	17 (5.7%)	7 (2.3%)	1 (0.3%)
Chewing gum, candies, and sweets (*n* = 111)	37	11	13	13	12	17	7	1
Desserts and other sweets (*n* = 55)	12	11	31	1	0	0	0	0
Table-top sweeteners (*n* = 52)	28	22	2	0	0	0	0	0
Chocolates (*n* = 46)	17	24	4	1	0	0	0	0
Jams (*n* = 35)	3	20	4	8	0	0	0	0
Non-alcoholic beverages (*n* = 266; 21.5%)	113 (42.5%)	100 (37.6%)	51 (19.2%)	2 (0.75%)	0	0	0	0
Diet Soft Drinks (*n* = 91)	14	43	34	0	0	0	0	0
Sugar Soft Drinks (*n* = 90)	61	20	7	2	0	0	0	0
Other non-alcoholic drinks (*n* = 32)	9	14	9	0	0	0	0	0
Fruit juices and nectars (*n* = 30)	15	14	1	0	0	0	0	0
Energy drinks (*n* = 14)	7	7	0	0	0	0	0	0
Coffee and herbal teas (*n* = 6)	6	0	0	0	0	0	0	0
Sport drinks (*n* = 3)	1	2	0	0	0	0	0	0
Cereals and grains (*n* = 239; 19.3%)	196 (82%)	35 (14.6%)	8 (3.3%)	0	0	0	0	0
Bakery and pastry (*n* = 186)	151	30	5	0	0	0	0	0
Breakfast cereal and cereal bars (*n* = 48)	41	4	3	0	0	0	0	0
Bread (*n* = 5)	4	1	0	0	0	0	0	0
Milk and dairy products (*n* = 169; 13.6%)	30 (17.7%)	122 (72.2%)	8 (5%)	8 (5%)	1 (0.6%)	0	0	0
Yogurt and fermented milks (*n* = 126)	20	99	7	0	0	0	0	0
Other dairy products (*n* = 41)	10	21	1	8	1	0	0	0
Cheese (*n* = 2)	0	2	0	0	0	0	0	0
Food supplements and substitutes (*n* = 109; 8.8%)	79 (72.5%)	26 (24%)	4 (3.6%)	0	0	0	0	0
Meat and meat products (*n* = 73; 6%)	73 (100%)	0	0	0	0	0	0	0
Ready to eat meals (*n* = 28; 2.2%)	28 (100%)	0	0	0	0	0	0	0
Appetizers (*n* = 19; 1.5%)	19 (100%)	0	0	0	0	0	0	0
Sauces and condiments (*n* = 11; 1%)	9 (82%)	2 (8%)	0	0	0	0	0	0
Canned fruit (*n* = 9; 0.7%)	2 (22%)	6 (66%)	1 (11%)	0	0	0	0	0
Fish and shellfish (*n* = 9; 0.7%)	9 (100%)	0	0	0	0	0	0	0
Alcoholic beverages (*n* = 6; 0.5%)	0	5 (83.3%)	1 (16.6%)	0	0	0	0	0
Vegetables (*n* = 1; 0.08%)	0	1 (100%)	0	0	0	0	0	0
*n* total products = 1,238	655	385	127	33	13	17	7	1
% of each combination of LNCS	52.9	31.1	10.3	2.7	1.1	1.4	0.6	0.1

The main food and beverage subgroups including LNCS were bakery and pastry (*n* = 186; 15%), yogurt and fermented milks (*n* = 126; 10.1%), chewing gums, candies, and sweets (*n* = 111; 9%), food supplements and substitutes (*n* = 109; 8.8%), diet soft drinks (*n* = 91; 7.3%), sugar soft drinks (*n* = 90; 7.2%) and meat and meat products (*n* = 73; 6%), which together accounted for more than 60% of total products.

We found that 52.9% of products declared the use of only one LNCS, while 31.1% declared two LNCS and 10.3% declared three LNCS. The major groups that included just one LNCS were cereals and grains (82%), non-alcoholic beverages (42.5%), and sugars and sweets (32.4%), expressed as within-group proportion. The main groups that included two LNCS were milk and dairy products (72.2%), canned fruit (66%), and non-alcoholic beverages (37.6%). Non-alcoholic beverages (19.2%) and sugars and sweets (18%) showed the highest proportion of products including three LNCS. We recorded combinations of up to eight different LNCS, but these were minor and found in the chewing gum, candies, and sweets subgroup. The comprehensive distribution of the specific types and combinations of LNCS declared across the assessed food groups and subgroups can be retrieved from the Supplementary Material section, Supplementary Table 1.

Table 2 shows the different types of LNCS declared among the food and beverage subgroups included in the database. Globally, we observed that sorbitol, sucralose, and acesulfame K were the most declared LNCS with 423 (19.5%), 421 (19.5%), and 415 (19.2%) label declarations, respectively. Sugars and sweets, which were the main group found in our survey, declared the use of a wide variety of LNCS across their subgroups: chocolates mainly include sorbitol and steviol glycosides; jams include sucralose, steviol glycosides, and maltitol; chewing gum, candies and sweets primarily declared sorbitol, acesulfame, K aspartame, and maltitol. Table-top sweeteners identified included steviol glycosides, saccharine, and cyclamate, among others. Desserts (i.e., gelatin) were comprised mainly of acesulfame K, sucralose, sorbitol, and maltitol and were the most widely used LNCS in the cereal and grain group. Within the non-alcoholic beverages group, acesulfame K and sucralose were the most frequent, especially in sugar soft drinks, while diet soft drinks declared the use of both but also the presence of aspartame and cyclamate. Meat and meat products declared only one type of LNCS, Sorbitol. Within the milk and dairy products group, where yogurt and fermented milk were the major subgroups, the mainly declared LNCS were acesulfame K, sucralose, and aspartame. Ready-to-eat meals, as well as appetizers and supplements and meal replacements, included sorbitol, and the latter also declared mainly sucralose, acesulfame K, and steviol glycosides. The minor group of LNCS was taumatine and (0.1%) and neotame (0.1%) that were present in table-top sweeteners and yogurt and fermented milk, respectively.

**Table 2:** Declared types and distribution (n) of LCNS across assessed food groups and subgroups available in the Spanish market.

	**Sorbitol** **(E-420)n**	**Mannitol** **(E-421)n**	**Acesulfame K (E-950)n**	**Aspartame (E-951)n**	**Cyclamate (E-952) n**	**Isomalt (E-953)n**	**Saccharine (E-954)n**	**Sucralose (E-955)n**	**Taumatine (E-957) n**	**Neohesperidine DC (E-959)n**	**Steviol glycosides (E-960) n**	**Neotame (E-961)n**	**Maltitol (E-965) n**	**Lactitol (E-966)n**	**Xylitol (E-967) n**	**Eritritol (E-968)n**
**Sugars and sweets (** ***n*** **=** **299)**																
Chewing gum, candies, and sweets (*n* = 111)	91	31	74	62	0	44	2	39	0	3	7	0	49	0	29	0
Desserts and other sweets (*n* = 55)	11	1	16	6	0	1	0	14	0	0	0	0	5	0	0	0
Tabletop sweeteners (*n* = 52)	0	0	4	7	15	0	16	6	2	0	22	0	0	0	3	5
Chocolates (*n* = 46)	0	0	7	4	0	3	0	3	0	1	17	0	39	2	0	2
Jams (*n* = 35)	12	0	8	0	0	4	0	28	0	0	19	0	16	0	0	0
**Non-alcoholic beverages (** ***n*** **=** **266)**																
Diet soft drinks (*n* = 91)	0	0	67	40	31	0	10	48	0	2	5	0	0	0	0	0
Sugar soft drinks (*n* = 90)	0	1	25	7	10	0	6	59	0	3	22	0	0	0	0	0
Other non-alcoholic drinks (*n* = 32)	0	0	12	1	4	0	3	13	0	0	6	0	0	0	0	0
Fruit juices and nectars (*n* = 30)	0	0	14	1	5	0	4	20	0	0	7	0	1	0	0	0
Energy drinks (n = 14)	0	0	9	2	0	0	0	14	0	0	0	0	0	0	0	0
Coffee and herbal teas (*n* = 6)	0	0	0	1	0	0	0	3	0	0	2	0	0	0	0	0
Sport drinks (*n* = 3)	0	0	2	0	0	0	0	3	0	0	0	0	0	0	0	0
**Cereals and grains (** ***n*** **=** **239)**																
Bakery and pastry (*n* = 186)	120	0	13	0	1	12	1	8	0	0	1	0	82	1	1	2
Breakfast cereal and cereal bars (*n* = 48)	24	0	3	0	0	1	0	4	0	0	1	0	14	0	2	0
Bread (*n* = 5)	4	0	1	1	0	0	0	0	0	0	0	0	0	0	0	0
**Milk and dairy products (** ***n*** **=** **169)**																
Yogurt and fermented milks (*n* = 126)	0	0	105	30	0	0	0	87	0	0	14	3	0	0	0	0
Other dairy products (*n* = 41)	10	1	31	11	6	0	4	23	0	2	5	0	21	10	0	2
Cheese (*n* = 2)	0	0	2	1	0	0	0	1	0	0	0	0	0	0	0	0
Food supplements and substitutes (*n* = 109)	36	4	18	8	7	2	5	37	0	0	15	0	10	0	6	1
**Meat and meat products (** ***n*** **=** **73)**																
Sausages and other meat products (*n* = 70)	70	0	0	0	0	0	0	0	0	0	0	0	0	0	0	0
Meat (*n* = 3)	3	0	0	0	0	0	0	0	0	0	0	0	0	0	0	0
Ready-to-eat meals (*n* = 28)	23	1	0	1	0	0	0	0	0	0	0	0	0	0	0	0
Appetizers (*n* = 19)	8	2	0	3	0	4	0	0	0	0	0	0	2	0	0	0
Sauces and condiments (*n* = 11)	1	0	0	1	2	0	4	3	0	2	0	0	0	0	0	0
Fish and shellfish (*n* = 9)	9	0	0	0	0	0	0	0	0	0	0	0	0	0	0	0
Canned fruit (*n* = 9)	0	0	0	0	0	0	1	8	0	0	0	0	0	0	0	0
Alcoholic beverages (*n* = 6)	0	0	4	0	2	0	0	0	0	0	0	0	0	0	0	0
Vegetables (*n* = 1)	1	0	0	1	0	0	0	0	0	0	0	0	0	0	0	0
*n* total products = 1,238/LNCS total declarations	423	41	415	188	83	71	56	421	2	13	143	3	239	13	41	12
LNCS % within total declarations (*n* = 2,164)	19.5%	2%	19.2%	8.7%	3.8%	3.3%	2.6%	19.5%	0.1%	0.6%	6.6%	0.1%	11%	0.6%	2%	0.6%

***n**, number of products included in each subgroup and types of LNCS are declared on labels of the products. One or more LNCS could be declared in products from each subgroup. For further description, refer to Supplementary Table 1*.

## Discussion

In a global level, when comparing the food product database of 2019 with 2017, we currently observed that a higher number of food products included LNCS since there were more food products included in the webs of food retailers and, potentially, an increased number of reformulated products that have total or partially substituted added sugar contents.

Among the first studies that described LNCS consumption is the ANIBES Study (7), back in 2103, where food and beverage products consumed by participants were extracted from food records obtained from a three-day dietary record using a tablet device. Label data from 1,164 products of different brands were collected and reviewed for the content of LNCS. The results showed the diversity of food groups including these ingredients that the population consumed but failed to describe the market availability.

Then, in 2017, we started the compilation of a food composition database that included all products declaring LNCS in their ingredients lists (8). The present study follows the same methodology and, therefore, comparisons might be made with great precision. Concerning major food groups available, soft drinks, fruit juices and nectars, yogurts and fermented milk, and chewing gum and candies were coincident in their distribution. In turn, the new update shows a major proportion of bakery and pastry products (16% *vs*. 8%), supplement and meal replacers (9% *vs*. 4%), breakfast cereals and bars (3% *vs*. 1%), and ready-to-eat meals (2% *vs*. 1%). The food and beverage groups and subgroups containing LNCS are available more in number in the Spanish market, new food groups found in the present survey included meat and meat products (*n* = 73), fish and shellfish (*n* = 9), alcoholic beverages (*n* = 6), coffee and herbal teas (*n* = 6), and bread products (*n* = 5) and cheese (*n* = 2).

When assessing the types and combinations of LNCS added, the previous database showed that the most frequent combination of LNCS found was acesulfame K with sucralose (22%; *n* = 81), followed by individual addition of sucralose (13%; *n* = 49) and sorbitol (9%; *n* = 34), whereas mannitol, xylitol, neohesperidine DC, and lactitol were minor (≤ 1%). Similarly, in the present update, we found that the most frequent combination was acesulfame K with sucralose. In addition, sorbitol (19.5%; *n* = 423), sucralose (19.5%; *n* = 421) and acesulfame K (19.2%; *n* = 415) were the most declared LNCS. In addition, mannitol and xylitol increased their frequency of addition. We found that 52.9% of products from the current database declared the use of one LNCS, while 31.1% declared two LNCS and 10.3% declared three LNCS. The higher number of combinations determines that because a variety of LNCS is used, lower quantities of each one might be needed to be added, and this leads to the possibility of not exceeding the acceptable daily intake levels (ADI) (regarded as a safety threshold). In contrast, the larger availability of different types of foods containing LNCS reinforces the importance of continuous monitoring of the food consumption by the Spanish Population to follow up the dietary model weighing benefits and risks in the new post-reformulation era.

The contribution of these food and beverage groups containing LNCS to the daily total TE of the Spanish diet might be studied by reviewing the findings from the ANIBES study (16) that assessed the dietary intake of free and intrinsic sugars and their major food sources in the Spanish Population aged 9–75 years. The median total daily sugar consumption in the Spanish population was 71.5 g, contributing 17% of the TE intake. Of these, free sugars (i.e., added) accounted for 28.8 g (0.0–189.8 g; min–max) and 7.3% of TE. Therefore, the potential substitution of added sugars with LNCS, as shown within the major food and beverage groups, found in the present study could represent a decrease of up to 7.3% of TE, assuming that all sugar contents from these products were replaced by LNCS. The ANIBES findings indicate that the major sources contributing to free sugar intakes were sugar soft drinks (25.5%), sugar (17.8%), bakery and pastry items (15.2%), chocolates (11.4%), yogurt and fermented milk (6.44%), other dairy products (5.99%), jams (3.58%), juices and nectars (2.91%), and breakfast cereals and cereal bars (2.78%). Within these food and beverage products, we found in the present study that soft drinks, bakery and pastry, chocolates, and yogurt and fermented milk included LNCS in a higher percentage. Interestingly, a report from the Spanish Ministry of Agriculture, Fisheries, and Food in 2019 (17) showed that consumption of LNCS increased 1.6% from 2018 but did not give any further information on types of LNCS or the food groups to which they are added.

It is well-known that the packaged food and beverage segments of the food supply are dynamic and are characterized by continuous change and turnover as new products are introduced and less-favored products are removed from the retail market (18). These frequent changes require food composition databases to be continuously updated, however, so far LNCS are not included in most food composition databases worldwide, Spain is no exception. In addition, it is noteworthy that there is a fewer number of European studies assessing the presence of LNCS in food products.

In an Italian study (19) that collected 326 products containing LNCS from a label survey, non-alcoholic beverages, table-top sweeteners, and food supplements were the major contributors for almost all sweeteners. The most consumed sweeteners were acesulfame K, aspartame, and cyclamate. A study led in Belgium that was also conducted by a label survey between December 2009 and February 2010 (20), included 270 products, found that aspartame was the most frequently used LNCS (34%) as a single sweetener or primarily in combination with acesulfame K, followed by saccharine (24%), and cyclamate (22%). In addition, it was observed that the most important group were beverages, including non-alcoholic drinks (44%) and beers (12%), which, together, represented more than half of the total supply of sweetened foods. Sweets accounted for 12%, chewing gums for 4% and a noteworthy result was that only one cereal product contained LNCS as opposed to this study results, where cereals and grains were 19% of total products surveyed (20).

In countries like Chile, where heavy regulations to label high sugar content products are in place, the use of LNCS has increased as a part of food reformulation by manufacturers (21). Researchers highlight that there might be overuse of these additives and that it might represent a risk for some key populations, such as children, but a recent study (22) showed that this group does not exceed the ADI of any of the six LNCS authorized in Chile. The major food groups containing LNCS were non-alcoholic beverages (38.2%), 28.8% dairy products, 15.6% sweets and other desserts, 14.5% cereal products, and 2.9% processed fruits. Regarding the number of LNCSs present in these products, 42% contained only one LNCS, 48.1% had two LNCS, 8.6% had three LNCS, and only 1.4% had four LNCS (21). As for the types of LNCS from the Chilean study, sucralose and steviol glycosides were the most widely used LNCS, these sweeteners are present, either alone or mixed with other, in 73.5 and 39.7% of the LNCS-containing products, respectively, while the use of saccharin and cyclamate was low (21).

It seems evident from these data that LNCS distribution across food and beverage groups and the type of LNCS used is highly variable depending on the country studied. In addition, as reformulation practices are increasing to comply with plans promoting the reduction of added sugars, it could be relevant that EU regulations mandate the quantities of LNCS to be included in the ingredient composition to evaluate the potential level of ADI achieved by target populations, such as children.

At present, there is a controversy regarding the potential health benefits and risks associated with the use of LNCS. In a recent review by our group, we found a limited number of representative studies on the consumption of LNCS and its effect on health (3). However, these mostly indicate that the consumption of LNCS can be a useful tool along with other nutritional strategies in the treatment of overweight, obesity (23), diabetes (24), and the prevention of caries (25), when used appropriately in the context of a balanced diet and physical activity. However, it is necessary to be cautious with the consumption of certain sweeteners since the effects of LNCS on the intestinal microbiota (26) or its effect on premature deliveries (27), among others, have not been fully elucidated. Further studies should be undertaken to clarify the safety and value of sweeteners as food additives in the medium/long-term, in a model of increasing consumption as a consequence of the reformulation of many foods.

### Strengths and Limitations

Among the main strengths of this study, we may highlight the representativity of food and beverage products assessed, as the main Spanish retailer online platforms were scanned for LNCS containing products. In addition, the detailed description per food group and subgroup enables a further assessment of the intakes of the population. A possible limitation of the present work is the potential lack of updated information on the label of the product available from these retailers: availability of new products, reformulation by manufacturers, and elimination of others might hinder the comprehensive collection of data. Furthermore, to date, data regarding LNCS concentration among different food groups remain unavailable, given that manufacturers are not mandated to declare this information. Therefore, the actual content of LNCS (and not only presence) across food and beverage products remains unknown in our country. One major limitation of this study was the inability to evaluate the actual content of LNCS to be able to estimate dietary intakes. In this regard, biomarker analysis could be combined with dietary assessment as suggested by Logue et al. (28), who used a novel urinary biomarker approach for the determination of up to five LNCS to characterize consumption in adults.

## Conclusion

This study showed that the variety of LNCS in the present Spanish diet increased when compared with the previous cross-survey in 2017, where mainly soft drinks, fruit juices and nectars, yogurts and fermented milks, and chewing gum and candies contained LNCS; as they are now included in further subgroups such as meat and meat products, fish and shellfish, alcoholic beverages, coffee and herbal teas, bread products and cheese. The number and type of LNCS combinations have also improved, and this is remarkable because manufacturers can diversify the use of LNCS and decrease the amounts used in each of them. In addition, data concerning LNCS contents and presence in food and beverages are still not compiled in the food composition databases, and they should be periodically updated to enable their assessment and monitoring in nutritional surveys. As a result, further studies are needed in the future to continuously evaluate the evolution of the presence of LNCS in the Spanish diet. In addition, studies assessing the content of LNCS in foods are required, using the present investigation as a guide on which compounds should be investigated in food groups.

## Data Availability Statement

The raw data supporting the conclusions of this article will be made available by the authors, without undue reservation.

## Author Contributions

BG-F and RU created the database. GV-M, RU, MS-V, and TP performed analyses and wrote the first draft of the manuscript. All authors contributed to read and approved the final manuscript.

## Conflict of Interest

The authors declare that the research was conducted in the absence of any commercial or financial relationships that could be construed as a potential conflict of interest.

## Publisher's Note

All claims expressed in this article are solely those of the authors and do not necessarily represent those of their affiliated organizations, or those of the publisher, the editors and the reviewers. Any product that may be evaluated in this article, or claim that may be made by its manufacturer, is not guaranteed or endorsed by the publisher.
