# Supplementary material for: Updated Database and Trends of Declared Low- and No-Calorie Sweeteners From Foods and Beverages Marketed in Spain
Source: Front Nutr. 2021 Jul 29;8:670422. doi: 10.3389/fnut.2021.670422 (PMC8358294; doi:10.3389/fnut.2021.670422)
Supplement: Supplementary file 1 [file Table_1.DOCX]

| **Supplementary Table 1.** Number and type of low and no-calorie sweeteners (LNCS) as declared in the ingredient’s list of food group and subgroup from the Spanish market. | | | |
| --- | --- | --- | --- |
| **Number of LNCS declared in the ingredient list** | **Type of LNCS**  **(number of products)** | **Food groups containing each type of individual or combined LNCS**  **(number of products)** | **Total foods** |
| 1 | Sorbitol  (*n*=140) | Appetizers (*n*= 8), Chewing gum, candies, and sweets (*n*= 35), Dessert and other sweets (*n*= 10), Meat (*n*= 3), Sausages and other meat products (*n*= 70), Bread (*n*= 4), Bakery and pastry (*n*= 107), Breakfast cereal and cereal bars (*n*= 23), Other dairy products (*n*= 4), Fish and shellfish (*n*=9), Ready to eat meals (*n*= 26), Sauces and condiments (*n*= 1), Food supplements and substitutes (*n*= 30) | ***n*= 655**  **(52.9 %)** |
|  | Mannitol  (*n=* 1) | Ready to eat meals (*n*= 1) |  |
|  | Acesulfame K  (*n=* 17) | Appetizers (*n*= 2), Fruit juices and nectars (*n*= 1), Bakery and pastry (*n*= 1), Breakfast cereal and cereal bars (n= 11), Food supplements and substitutes (*n*= 2) |  |
|  | Aspartame  (*n=* 13) | Appetizers (*n*= 3), Table-top sweeteners (*n*= 3), Coffee and herbal teas (*n*= 1), Other dairy products (*n*= 3), Ready to eat meals (*n*= 1), Sauces and condiments (*n*= 1), Food supplements and substitutes (*n*= 1) |  |
|  | Isomalt  (*n=* 3) | Chewing gum, candies, and sweets (*n*= 1), Food supplements and substitutes (*n*= 2) |  |
|  | Saccharine  (*n=* 7) | Appetizers (*n*= 4), Table-top sweeteners (*n*= 1), Sauces and condiments (*n*= 2) |  |
|  | Sucralose  (*n=* 119) | Table-top sweeteners (*n*= 5), Dessert and other sweets (*n*= 2), Coffee and herbal teas (*n*= 3), Sugar Soft Drinks (*n*= 39), Diet Soft Drinks (*n*= 10), Sport drinks (*n*= 1), Energy drinks (*n*= 7), Fruit juices and nectars (*n*= 7), Other non-alcoholic drinks (*n*= 4), Canned fruit (*n*= 2), Other dairy products (*n*= 2), Yogurt and fermented milks (*n*= 7), Sauces and condiments (*n*= 4), Food supplements and substitutes (*n*= 26) |  |
|  | Steviol glycosides  (*n*= 89) | Chocolates (*n*= 1), Jams (*n*= 3), Table-top sweeteners (*n*= 16), Coffee and herbal teas (*n*= 2), Sugar Soft Drinks (*n*= 22), Diet Soft Drinks (*n*= 4), Fruit juices and nectars (*n*= 7), Other non-alcoholic drinks (*n*= 5), Other dairy products (*n*= 1), Yogurt and fermented milks (*n*= 13), Sauces and condiments (*n*= 1), Food supplements and substitutes (*n*= 14) |  |
|  | Maltitol  (*n*= 72) | Appetizers (*n*= 2), Chocolates (*n*= 16), Chewing gum, candies, and sweets (*n*= 1), Bakery and pastry (*n*= 43), Breakfast cereal and cereal bars (*n*= 7), Food supplements and substitutes (*n*= 3) |  |
|  | Lactitol  (*n*= 1) | Food supplements and substitutes (*n*= 1) |  |
|  | Xylitol  (*n*= 3) | Table-top sweeteners (*n*= 3) |  |
| **2** | Steviol glycosides  Maltitol  (*n*= 25) | Chocolates (*n*= 18), Chewing gum, candies, and sweets (*n*= 6), Breakfast cereal and cereal bars (*n*= 1) | ***n*= 385**  **(31.1 %)** |
|  | Steviol glycosides  Eritritol  (*n*= 4) | Chocolates (*n*= 2), Other dairy products (*n*= 2) |  |
|  | Steviol glycosides  Lactitol  (*n*= 1) | Chocolates (*n*= 1) |  |
|  | Acesulfame K  Aspartame  (*n*= 60) | Chocolates (*n*= 2), Table-top sweeteners (*n*= 3), Dessert and other sweets (*n*= 1), Sugar Soft Drinks (*n*= 2), Diet Soft Drinks (*n*= 15), Energy drinks (*n*= 2), Fruit juices and nectars (*n*= 1), Other non-alcoholic drinks (*n*= 1), Bread (*n*= 1), Bakery and pastry (*n*= 1), Yogurt and fermented milks (*n*= 26), Cheese (*n*= 1), Food supplements and substitutes (*n*=4) |  |
|  | Acesulfame K  Sucralose  (*n*= 162) | Chocolates (*n*= 1), Dessert and other sweets (*n*= 10), Sugar Soft Drinks (*n*= 13), Diet Soft Drinks (*n*= 22), Sport drinks (*n*= 2), Energy drinks (*n*= 5), Fruit juices and nectars (*n*= 10), Other non-alcoholic drinks (*n*= 8), Canned fruit (*n*= 2), Other dairy products (*n*= 11), Yogurt and fermented milks (*n*= 71), Cheese (*n*= 1), Food supplements and substitutes (*n*= 6) |  |
|  | Sucralose  Maltitol  (*n*= 30) | Jams (*n*= 15), Bakery and pastry (*n*= 9), Breakfast cereal and cereal bars (*n*= 1), Canned fruit (*n*= 3) Other dairy products (*n*= 1), Food supplements and substitutes (*n*= 1) |  |
|  | Sucralose  Steviol glycosides  (*n*= 8) | Jams (*n*= 5), Diet Soft Drinks (*n*= 1), Yogurt and fermented milks (*n*= 1), Food supplements and substitutes (*n*= 1) |  |
|  | Aspartame  Xylitol  (*n*= 2) | Chewing gum, candies, and sweets (*n*= 2) |  |
|  | Isomalt  Maltitol  (*n*= 9) | Chewing gum, candies, and sweets (*n*= 1), Bakery and pastry (*n*= 8) |  |
|  | Acesulfame K  Isolmalt  (*n*= 2) | Chewing gum, candies, and sweets (*n*= 2) |  |
|  | Cyclamate  Saccharine  (*n*= 30) | Table-top sweeteners (*n*= 13), Sugar Soft Drinks (*n*= 2), Diet Soft Drinks (*n*= 2), Other non-alcoholic drinks (*n*= 3), Low alcohol content beverages (*n*= 2), Canned fruit (*n*= 1), Other dairy products (*n*= 1), Sauces and condiments (n= 2), Food supplements and substitutes (n= 4) |  |
|  | Steviol glycosides  Eritritol  (*n*= 2) | Table-top sweeteners (n= 1), Bakery and pastry (n= 1) |  |
|  | Steviol glycosides  Xylitol  (*n*= 4) | Table-top sweeteners (n= 4) |  |
|  | Acesulfame K  Steviol glycosides  (*n*= 2) | Table-top sweeteners (*n*= 1), Other non-alcoholic drinks (*n*= 1) |  |
|  | Acesulfame K  Cyclamate  (*n*= 6) | Other non-alcoholic drinks (*n*= 1), Low alcohol content beverages (*n*= 3), Food supplements and substitutes (*n*= 2) |  |
|  | Cyclamate  Sucralose  (*n*= 7) | Sugar Soft Drinks (*n*= 3), Diet Soft Drinks (*n*= 3), Fruit juices and nectars (*n*= 1) |  |
|  | Acesulfame K  Taumatine  (*n*= 1) | Fruit juices and nectars (*n*= 1) |  |
|  | Acesulfame K  Saccharine  (*n*= 1) | Fruit juices and nectars (*n*= 1) |  |
|  | Sorbitol  Maltitol  (*n*= 11) | Bakery and pastry (*n*= 3), Other dairy products (*n*= 6), Food supplements and substitutes (*n*= 2) |  |
|  | Maltitol  Eritritol  (*n*= 1) | Bakery and pastry (*n*= 1) |  |
|  | Acesulfame K Maltitol  (*n*= 6) | Bakery and pastry (*n*= 5), Breakfast cereal and cereal bars (*n*= 1) |  |
|  | Acesulfame K  Lactitol  (*n*= 1) | Bakery and pastry (*n*= 1) |  |
|  | Aspartame  Isomalt  (*n*= 1) | Bakery and pastry (*n*= 1) |  |
|  | Isomalt  Sucralose  (*n*= 1) | Breakfast cereal and cereal bars (*n*= 1) |  |
|  | Sucralose  Neotame  (*n*= 1) | Yogurt and fermented milks (*n*= 1) |  |
|  | Mannitol  Xylitol  (*n*= 4) | Food supplements and substitutes (*n*= 4) |  |
|  | Xylitol  Eritritol  (*n*= 1) | Food supplements and substitutes (*n*= 1) |  |
|  | Sorbitol  Sucralose  (*n*= 1) | Food supplements and substitutes (*n*= 1) |  |
|  | Sorbitol  Aspartame  (*n*= 1) | Vegetables (*n*= 1) |  |
| **3** | Isomalt  Sucralose  Maltitol  (*n*= 2) | Chocolates (*n*=1), Bakery and pastry (*n*= 1) | ***n*= 127**  **(10.3 %)** |
|  | Acesulfame K  Aspartame  Maltitol  (*n*= 36) | Chocolates (*n*= 2), Dessert and other sweets (*n*= 31), Food supplements and substitutes (*n*= 3) |  |
|  | Acesulfame K  Maltitol  Lactitol  (*n*=1) | Chocolates (*n*= 1) |  |
|  | Sorbitol  Sucralose  Esteviol glycosides  (*n*= 4) | Jams (*n*= 4) |  |
|  | Sorbitol  Acesulfame K  Sucralose  (*n*=3) | Chewing gum, candies, and sweets (*n*= 3) |  |
|  | Acesulfame K  Aspartame  Isomalt  (*n*= 5) | Chewing gum, candies, and sweets (*n*= 5) |  |
|  | Acesulfame K  Isomalt  Sucralose  (*n*= 1) | Chewing gum, candies, and sweets (*n*= 1) |  |
|  | Acesulfame K  Isomalt  Neosperidine DC  (*n*= 3) | Chewing gum, candies, and sweets (*n*= 3) |  |
|  | Mannitol  Steviol glycosides  Maltitol  (*n*= 1) | Chewing gum, candies, and sweets (*n*= 1) |  |
|  | Cyclamate  Saccharine  Taumatine  (*n*= 2) | Table-top sweeteners (*n*= 2) |  |
|  | Acesulfame K  Cyclamate  Saccharine  (*n*= 4) | Sugar Soft Drinks (*n*= 1), Low alcohol content beverages (*n*= 1), Other dairy products (*n*= 2) |  |
|  | Mannitol  Acesulfame K  Sucralose  (*n*= 1) | Sugar soft drinks (*n*= 1) |  |
|  | Acesulfame K  Aspartame  Neosperidine DC  (*n*= 3) | Sugar soft drinks (*n*= 3) |  |
|  | Acesulfame K  Cyclamate  Sucralose  (*n*= 4) | Sugar soft drinks (*n*= 2), Diet soft drinks (*n*= 1), Other dairy products (*n*= 1) |  |
|  | Acesulfame K  Aspartame  Sucralose  (*n*= 12) | Diet soft drinks (*n*= 7), Yogurt and fermented milk (*n*= 5) |  |
|  | Aspartame  Cyclamate  Sucralose  (*n*= 2) | Diet soft drinks (*n*= 2) |  |
|  | Acesulfame K  Sucralose  Neosperidine DC  (*n*= 2) | Diet soft drinks (*n*= 2) |  |
|  | Acesulfame K  Aspartame  Cyclamate  (*n*= 17) | Diet soft drinks (*n*= 17) |  |
|  | Cyclamate  Saccharine  Sucralose  (*n*= 1) | Diet soft drinks (*n*= 1) |  |
|  | Acesulfame K  Cyclamate  Saccharine  (*n*= 3) | Diet soft drinks (*n*= 3) |  |
|  | Aspartame  Cyclamate  Saccharine  (*n*= 1) | Diet soft drinks (*n*= 1) |  |
|  | Cyclamate  Saccharine  Maltitol  (*n*= 1) | Fruit juices and nectars (*n*= 1) |  |
|  | Acesulfame K  Steviol glycosides  Maltitol  (*n*= 1) | Bakery and pastry (*n*= 1) |  |
|  | Sorbitol  Isomalt  Maltitol  (*n*= 2) | Bakery and pastry (*n*= 2) |  |
|  | Sorbitol  Acesulfame K  Sucralose  (*n*= 1) | Bakery and pastry (*n*= 1) |  |
|  | Sucralose  Maltitol  Xylitol  (*n*= 2) | Breakfast cereal and cereal bars (*n*= 2) |  |
|  | Sorbitol  Acesulfame K  Maltitol  (*n*= 1) | Breakfast cereal and cereal bars (*n*= 1) |  |
|  | Sucralose  Steviol glycosides  Maltitol  (*n*= 1) | Canned fruit (*n*= 1) |  |
|  | Acesulfame K  Neosperidine DC  Steviol glycosides  (*n*= 1) | Other dairy products (*n*= 1) |  |
|  | Acesulfame K  Sucralose  Maltitol  (*n*= 4) | Other dairy products (*n*= 4) |  |
|  | Acesulfame K  Cyclamate  Neoesperidine DC  (*n*= 1) | Other dairy products (*n*= 1) |  |
|  | Acesulfame K  Sucralose  Neosperidine DC  (*n*= 1) | Other dairy products (*n*= 1) |  |
|  | Acesulfame K  Sucralose  Neotame  (*n*= 2) | Yogurt and fermented milks (*n*= 2) |  |
|  | Sorbitol  Cyclamate  Saccharine  (*n*= 1) | Food supplements and substitutes (*n*= 1) |  |
| **4** | Acesulfame K  Isomalt  Neosperidine DC  Maltitol  (*n*= 1) | Chocolates (*n*= 1) | ***n*= 33**  **(2.7 %)** |
|  | Sorbitol  Acesulfame K  Sucralose  Steviol glycosides  (*n*= 8) | Jams (*n*= 8) |  |
|  | Sorbitol  Acesulfame K  Aspartame  Xylitol  (*n*= 1) | Chewing gum, candies, and sweets (*n*= 1) |  |
|  | Sorbitol  Mannitol  Acesulfame K  Aspartame  (*n*= 1) | Chewing gum, candies, and sweets (*n*= 1) |  |
|  | Acesulfame K  Aspartame  Isomalt  Maltitol  (*n*= 4) | Chewing gum, candies, and sweets (n= 3) Dessert and other sweets (*n*= 1) |  |
|  | Sorbitol  Acesulfame K  Aspartame  Sucralose  (*n*= 5) | Chewing gum, candies, and sweets (*n*= 5) |  |
|  | Acesulfame K  Isomalt  Saccharine  Maltitol  (*n*= 2) | Chewing gum, candies, and sweets (*n*= 2) |  |
|  | Acesulfame K  Isomalt  Sucralose  Maltitol  (*n*= 1) | Chewing gum, candies, and sweets (*n*= 1) |  |
|  | Acesulfame K  Aspartame  Cyclamate  Sucralose  (*n*= 1) | Sugar Soft Drinks (*n*= 1) |  |
|  | Acesulfame K  Aspartame  Cyclamate  Saccharine  (*n*= 1) | Sugar Soft Drinks (*n*= 1) |  |
|  | Acesulfame K  Sucralose  Maltitol  Lactitol  (*n*= 2) | Other dairy products (*n*= 2) |  |
|  | Acesulfame K  Aspartame  Maltitol  Lactitol  (*n*= 6) | Other dairy products (*n*= 6) |  |
| **5** | Sorbitol  Acesulfame K  Aspartame  Sucralose  Xylitol  (*n*= 1) | Chewing gum, candies, and sweets (*n*= 1) | ***n*= 13**  **(1.1 %)** |
|  | Sorbitol  Mannitol  Acesulfame K  Aspartame  Isomalt  (*n*= 1) | Chewing gum, candies, and sweets (*n*= 1) |  |
|  | Sorbitol  Mannitol  Acesulfame K  Aspartame  Xylitol  (*n*= 5) | Chewing gum, candies, and sweets (*n*= 5) |  |
|  | Sorbitol  Acesulfame K  Aspartame  Maltitol  Xylitol  (*n*= 1) | Chewing gum, candies, and sweets (*n*= 1) |  |
|  | Sorbitol  Acesulfame K  Aspartame  Isomalt  Maltitol  (*n*= 3) | Chewing gum, candies, and sweets (*n*= 3) |  |
|  | Acesulfame K  Aspartame  Isomalt  Maltitol  Xylitol  (*n*= 1) | Chewing gum, candies, and sweets (*n*= 1) |  |
|  | Mannitol  Aspartame  Cyclamate  Saccharine  Lactitol  (*n*= 1) | Other dairy products (*n*= 1) |  |
| **6** | Sorbitol  Mannitol  Acesulfame K  Aspartame  Maltitol  Xylitol  (*n*= 1) | Chewing gum, candies, and sweets (*n*= 1) | ***n*= 17**  **(1.4 %)** |
|  | Sorbitol  Mannitol  Acesulfame K  Aspartame  Sucralose  Xylitol  (*n*= 3) | Chewing gum, candies, and sweets (*n*= 3) |  |
|  | Sorbitol  Mannitol  Acesulfame K  Aspartame  Sucralose  Maltitol  (*n*= 1) | Chewing gum, candies, and sweets (*n*= 1) |  |
|  | Sorbitol  Acesulfame K  Isomalt  Sucralose  Maltitol  Xylitol  (*n*= 2) | Chewing gum, candies, and sweets (*n*= 2) |  |
|  | Sorbitol  Acesulfame K  Aspartame  Isomalt  Sucralose  Maltitol  (*n*= 1) | Chewing gum, candies, and sweets (*n*= 1) |  |
|  | Sorbitol  Acesulfame K  Aspartame  Isomalt  Sucralose  Maltitol  (*n*= 3) | Chewing gum, candies, and sweets (*n*= 3) |  |
|  | Sorbitol  Acesulfame K  Aspartame  Sucralose  Maltitol  Xylitol  (*n*= 4) | Chewing gum, candies, and sweets (*n*= 4) |  |
| **7** | Sorbitol  Mannitol  Acesulfame K  Aspartame  Isomalt  Sucralose  Maltitol  (*n*= 9) | Chewing gum, candies, and sweets (*n*= 8), Dessert and other sweets (*n*= 1) | ***n*= 7**  **(0.6 %)** |
|  | Sorbitol  Mannitol  Acesulfame K  Aspartame  Sucralose  Maltitol  Xylitol  (*n*= 5) | Chewing gum, candies, and sweets (*n*= 5) |  |
|  | Sorbitol  Mannitol  Acesulfame K  Aspartame  Isomalt  Maltitol  Xylitol  (*n*= 1) | Chewing gum, candies, and sweets *(n*= 1) |  |
| **8** | Sorbitol  Mannitol  Xylitol  Acesulfame K  Aspartame  Isomalt  Sucralose  Maltitol  (*n*= 1) | Chewing gum, candies, and sweets (*n*= 1) | ***n*= 1**  **(0.1 %)** |
